# Molecular species delimitation of marine trematodes over wide geographical ranges: *Schikhobalotrema* spp. (Digenea: Haplosplanchnidae) in needlefishes (Belonidae) from the Pacific Ocean and Gulf of Mexico

**DOI:** 10.1017/S0031182023001245

**Published:** 2024-02

**Authors:** Gerardo Pérez-Ponce de León, Brenda Solórzano-García, Daniel C. Huston, Berenit Mendoza-Garfias, Jhonatan Cabañas-Granillo, Scott C. Cutmore, Thomas H. Cribb

**Affiliations:** 1 Escuela Nacional de Estudios Superiores unidad Mérida, Universidad Nacional Autónoma de México, Tablaje Catastral No. 6998, Carretera Mérida-Tetiz Km. 4.5, Municipio de Ucú, 97357 Mérida, Yucatán, Mexico; 2 Australian National Insect Collection, National Research Collections Australia, CSIRO, PO Box 1700, Canberra, ACT 2601, Australia; 3 Instituto de Biología, Universidad Nacional Autónoma de México, Ap. Postal 70-153. C.P., 045 10 Mexico, DF, Mexico; 4 Queensland Museum, Biodiversity and Geosciences Program, South Brisbane, QLD 4101, Australia; 5 School of the Environment, The University of Queensland, St Lucia, QLD 4072, Australia

**Keywords:** Australia, Digenea, DNA, marine fish, Mexico, new species, species delineation

## Abstract

Geographical distribution plays a major role in our understanding of marine biodiversity. Some marine fish trematodes have been shown to have highly restricted geographical distributions, while some are known to occur over very wide ranges; however, very few of these wide distributions have been demonstrated genetically. Here, we analyse species of the genus *Schikhobalotrema* (Haplosplanchnidae) parasitizing beloniforms from the tropical west Pacific, the eastern Pacific and the Gulf of Mexico (GoM). We test the boundaries of these trematodes by integrating molecular and morphological data, host association, habitat of the hosts and geographical distribution, following a recently proposed and standardized delineation method for the recognition of marine trematode species. Based on the new collections, *Schikhobalotrema huffmani* is here synonymized with the type-species of the genus, *Schikhobalotrema acutum*; *Sch. acutum* is now considered to be widely distributed, from the GoM to the western Pacific. Additionally, we describe a new species, *Schikhobalotrema minutum* n. sp., from *Strongylura notata* and *Strongylura marina* (Belonidae) from La Carbonera coastal lagoon, northern Yucatán, GoM. We briefly discuss the role of host association and historical biogeography of the hosts as drivers of species diversification of *Schikhobalotrema* infecting beloniforms.

## Graphical abstract

**Figure:**
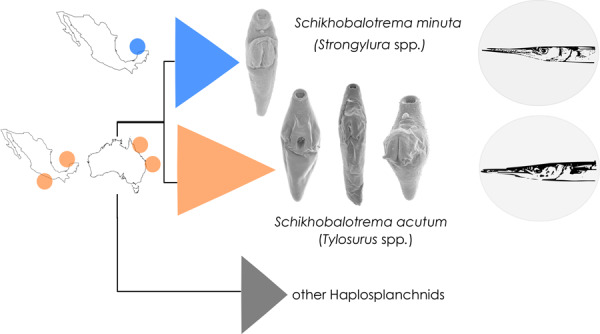

## Introduction

Trematodes of the family Haplosplanchnidae Poche, 1926 infect the digestive tract of a diverse array of marine fishes across the globe (Madhavi, 2005), with the group predominantly infecting herbivorous fishes (Huston *et al*., 2018). The traditional classification of the family considers species to be allocated into 4 subfamilies; however, based on recent phylogenetic analyses the subfamilies are not currently recognized by some authors (Huston *et al*., 2018). The family comprises 10 genera, of which the most speciose is *Schikhobalotrema* Skrjabin & Guschanskaja, 1955 with 26 valid species (Huston *et al*., 2017; WoRMS, 2023). *Schikhobalotrema* was proposed for *Deradena acutum* Linton, 1910, a parasite of 2 species of needlefish (Belonidae) from the Gulf of Mexico (GoM). While there have been a further 2 species described from belonids, like the rest of the Haplosplanchnidae, the members of *Schikhobalotrema* have overwhelmingly been described and reported from herbivorous fishes (particularly those of the families Acanthuridae, Hemiramphidae, Mugilidae, Pomacentridae and the scarine Labridae). In the most recent review of the genus, Huston *et al*. (2017) proposed a new species from 2 species of belonids off the eastern coast of Australia, and argued that the lack of molecular information for members of this genus, and the fact that the constituent species possess few complex morphological characters, makes the delimitation of species of *Schikhobalotrema* a difficult task.

While studying the trematode fauna of marine and estuarine fishes of Mexico, specimens of *Schikhobalotrema* were sampled from the intestines of belonids, namely *Tylosurus pacificus* (Steindachner) from off Chamela Bay and from off Barra de Coyuca, Acapulco on the eastern Pacific (EP) coast, *Tylosurus acus* off Celestún and *Strongylura marina* and *Strongylura notata* from La Carbonera coastal lagoon in northern Yucatán, GoM. In this study, we analyse the distribution of *Schikhobalotrema* species occurring in belonids over a wide geographical range that includes the tropical Indo-west Pacific (IWP), the tropical EP, and the GoM, using morphological and molecular data. Following the paradigm recently proposed for the recognition of marine fish trematode species by Bray *et al*. (2022) we document the convincingly wide distribution for the type-species and describe a new species of *Schikhobalotrema* from estuarine needlefishes of the genus *Strongylura* van Hasselt from La Carbonera coastal lagoon in Yucatán, GoM.

## Materials and methods

### Host collection and morphological study

Specimens of the Pacific agujon, *T. pacificus*, were obtained from commercial fisheries from 2 localities on the Pacific coast of Mexico: off Chamela Bay, Jalisco state in 1994; and off Barra de Coyuca, Acapulco, Guerrero state in 2018. Specimens of the agujon needlefish, *T. acus*, were obtained from a commercial fishery off the coast of Celestún, Yucatán in 2019, and specimens of the redfin needlefish, *St. notata* and the Atlantic needlefish, *St. marina* were collected from lagoons on the coast of Yucatán in the GoM in 2022. Fishes were dissected, the gastrointestinal tract removed, placed in Petri dishes with 0.85% saline solution and observed under a stereomicroscope. Trematodes morphologically identified as belonging to *Schikhobalotrema* were recovered alive and fixed in 2 different ways. Specimens from off Chamela Bay were fixed under slight coverslip pressure with Bouin's fluid and placed in vials with 70% ethyl alcohol (EtOH). Specimens from off Barra de Coyuca, Acapulco and from Yucatán were killed with nearly boiling 0.85% saline solution; some specimens were fixed without pressure in 10% formalin for morphological examination, and some were placed in vials with 100% EtOH for molecular analysis.

Specimens were stained with Mayer's paracarmine or Gomori's trichrome, dehydrated in a graded ethanol series, cleared in methyl salicylate and mounted as permanent slides in Canada balsam for morphological study. Specimens were observed using an Olympus BX51 light microscope equipped with differential interference contrast; drawings were made using a drawing tube attached to the same microscope. Measurements are expressed in micrometres, with the range followed by the mean in parentheses. Measurements of specimens from off Chamela Bay were not combined with those from the other specimens because the specimens were flattened. Specimens were deposited at the Colección Nacional de Helmintos (CNHE), Instituto de Biología, Mexico City. Two specimens from off Barra de Coyuca, Acapulco, 1 from off Celestún and 1 from La Carbonera lagoon were prepared for scanning electron microscopy (SEM). Specimens for SEM were dehydrated in a graded ethanol series, critical point dried and mounted on a strip of carbon conductive tape. Samples were sputter coated with gold and observed in a Hitachi Stereoscan Model SU1510 (Hitachi Ltd, Tokyo, Japan) at 10 kV.

Additionally, our analysis included specimens sampled from Australia in the form of paragenophores of samples incorporated in the description of *Schikhobalotrema huffmani* by Huston *et al*. (2017). Samples were collected from the hound needlefish, *Tylosurus crocodilus*, from off Lizard Island, Great Barrier Reef, Queensland, and from the stout longtom, *Tylosurus gavialoides*, from Moreton Bay, Queensland. In this study, novel SEM data of specimens of *Sch. huffmani* are presented to compare with samples obtained from the EP and GoM. Australian specimens for SEM were transferred from ethanol to hexamethyldisilazane, air-dried overnight and mounted on 12.5 mm pin-stubs using an adhesive carbon tab. Before performing SEM, specimens were coated with 15 nm of iridium with a Quorumtech Q150TS sputter coater. SEM images were obtained on a Hitachi SU3500 scanning electron microscope in secondary electron mode.

### DNA sequencing and phylogenetic analyses

Specimens preserved in 100% EtOH were digested overnight at 56°C in a solution containing 10 mm Tris-hydrochloric acid (pH 7.6), 20 mm sodium chloride, 100 mm Na_2_EDTA (pH 8.0), 1% sarkosyl and 0.1 mg mL^−1^ proteinase K. DNA was isolated from the supernatant using DNAzol (Molecular Research Center, Cincinnati, OH, USA) following the manufacturer's instructions. Fragments of the large (28S) and small (18S) subunits of ribosomal DNA were amplified *via* polymerase chain reaction using MyTaq™ DNA polymerase and the primers 502 (5′-CAA GTA CCG TGA GGG AAA GTT GC-3′) and 536 (5′-CAG CTA TCC TGA GGG AAA C-3′) (García-Varela and Nadler, 2005), and G18S4 (5′-GCT TGT CTC AAA GAT TAA GCC-3′) and 136 (5′-TGA TCC TTC TGC AGG TTC ACC TAC-3′) (Choudhury and Nadler, 2018), respectively. A fragment of the mitochondrial gene cytochrome oxidase C subunit 1 (*cox*1) was amplified with the primers Dig_CoxFa (5′-ATG ATW TTY TTY TTY YTD ATG CC-3′) and Dig_CoxR (5′-TCN GGR TGH CCR AAR AAY CAA AA-3′) (Wee *et al*., 2017). Contiguous sequences were assembled, and base-calling differences were resolved, using Geneious Pro 4.8.4 (Biomatters Ltd., Boston, USA). Sequences were deposited in the GenBank database.

DNA sequences were aligned using MUSCLE (Edgar, 2004) through the EMBL-EBI web interface (Madeira *et al*., 2019). Additional sequences of representatives of plagiorchiidans of the families Notocotylidae (*Catatropis indicus* Srivastava, 1935), Opisthotrematidae (*Lankatrema mannarense* Crusz & Fernand, 1954), Diplodiscidae (*Diplodiscus subclavatus* (Goeze, 1782)), Cladorchiidae (*Solenorchis travassosi* Hilmy, 1949), Psilostomidae (*Neopsilotrema lakotae* Kudlai, Pulis, Kostadinova & Tkach, 2016 and *Psilochasmus oxyurus* (Creplin, 1851) [Lühe, 1909]) and Echinostomatidae (*Echinostoma revolutum* (Fröhlich, 1802) Looss, 1899) were incorporated as outgroup taxa (Table 1). A substitution model was inferred using MrModeltest v. 2.3 (Nylander, 2004) following the Akaike's information criterion, obtaining GTR + I + G as the best model. Bayesian inference (BI) analyses were performed independently for each of the 3 genes. The 2 ribosomal genes were then concatenated, and a BI analysis was conducted to infer the interrelationships among the specimens sampled and other haplosplanchnid species available in GenBank. BI analysis was conducted using MrBayes v. 3.2.2 (Ronquist *et al*., 2012) on the CIPRES Science Gateway (Miller *et al*., 2010). The analysis included 2 simultaneous runs of Markov chain Monte Carlo, each for 4 million generations, sampling trees every 4000 generations, a heating parameter value of 0.2 and a ‘burn-in’ of 25%. A 50% majority-rule consensus tree was constructed from the post burn-in trees. BI outputs were imported to FigTree v. 1.4 (Rambaut, 2014) for graphical visualization and editing.

**Table 1.** GenBank accession numbers of DNA sequences used in phylogenetic analyses of members of the order Haplosplanchnata during this study

Taxon	Host family	Host species	28S	18S	Reference
Ingroup					
*Schikhobalotrema sparisomae* (Manter, 1937)	Mugilidae	*Liza aurata*	FJ211240	FJ211223	Blasco-Costa (2009)
*Schikhobalotrema* sp.	Labridae	*Scarus rivulatus*	AY222238	AJ287574	Olson *et al*. (2003); Cribb *et al.* (2001)
*Haplosplanchnus pachysomus* (Eysenhardt, 1829)	Mugilidae	*Liza ramado*	FJ211241	FJ211224	Blasco-Costa (2009)
	Mugilidae	*Liza engeli*	LK932149	LK932143	Besprozvannykh *et al*. (2016)
*Provitellotrema crenimugilis* Pan, 1984	Mugilidae	*Liza haematocheila*	LK932153–LK932154	LK932147–LK932148	Besprozvannykh *et al*. (2016)
*Haplosplanchnus purii* Srivastava, 1939	Mugilidae	*Mugil cephalus*	FJ211242	FJ211225	Blasco-Costa (2009)
*Trigonocephalotrema sohcahtoa* Huston, Cutmore & Cribb, 2018	Acanthuridae	*Zebrasoma scopas*	MG386261	MG386260	Hutson *et al*. (2018)
*Trigonocephalotrema euclidi* Huston, Cutmore & Cribb, 2018	Acanthuridae	*Naso unicornis*	MG386255	MG386254	Hutson *et al*. (2018)
*Trigonocephalotrema hipparchi* Huston, Cutmore & Cribb, 2018	Acanthuridae	*Naso brevirostris*	MG386258	MG386257	Hutson *et al*. (2018)
*Trigonocephalotrema* sp.	Acanthuridae	*Naso lituratus*	MG386264	MG386263	Hutson *et al*. (2018)
*Pseudohaplosplanchnus catbaensis* Atopkin, Besprozvannykh, Ha, Nguyen & Nguyen, 2020	Mugilidae	*Moolgarda seheli*	MT298959, MT298962	MT298954, MT298957	Atopkin *et al*. (2020)
*Hymenocotta mulli* Manter, 1961	Mugilidae	*Crenimugil crenilabis*	AY222239	AJ287524	Olson *et al*. (2003); Cribb *et al.* (2001)
*Schikhobalotrema acutum* (Linton, 1910)	Belonidae	*Tylosurus acus*	OR753897	OR753905	**This study**
	Belonidae	*Tylosurus pacificus*	OR753902–OR753904	OR753906	
*Schikhobalotrema acutum* (Linton, 1910) as *Schikhobalotrema huffmani* Huston, Cutmore & Cribb, 2017	Belonidae	*Tylosurus crocodilus*	KY852463	KY852461	Huston *et al.* (2017)
	Belonidae	*Tylosurus gavialoides*	KY852464	KY852462	
*Schikhobalotrema minuta* n. sp.	Belonidae	*Strongylura notata*	OR753898–OR753899 OR753901	OR753907–OR753908 OR753910	**This study**
	Belonidae	*Strongylura marina*	OR753900	OR753909	
Outgroup taxa					
*Catatropis indicus* Srivastava, 1935	Anatidae	*Cairina moschata*	AY222220	AY222114	Olson *et al*. (2003)
*Lankatrema mannarense* Crusz and Fernand, 1954	Dugongidae	*Dugong dugong*	AY222222	AY222116	Olson *et al*. (2003)
*Diplodiscus subclavatus* (Goeze, 1782)	Ranidae	*Rana ridibunda*	AY222212	AJ287502	Olson *et al*. (2003); Cribb *et al*. (2001)
*Solenorchis travassosi* Hylmi, 1949	Dugongidae	*Dugong dugong*	AY222213	AY222110	Olson *et al*. (2003)
*Psilochasmus oxyurus* Creplin, 1851	Anatidae	*Anas platyrhynchus*	AF151940	AY222135	Olson *et al*. (2003)

A neighbour-joining (NJ) tree was generated in MEGA v. 11 (Tamura *et al*., 2021) using the *cox*1 sequences of *Schikhobalotrema* species from Australia and Mexico, based on the Tamura–Nei model, gamma distribution rate and 500 bootstrap replicates. Genetic divergence (*P*-distance and number of nucleotide differences) between the new species and other haplosplanchnids was also calculated in MEGA.

## Results

### General phylogenetic results

**Figure 1.**
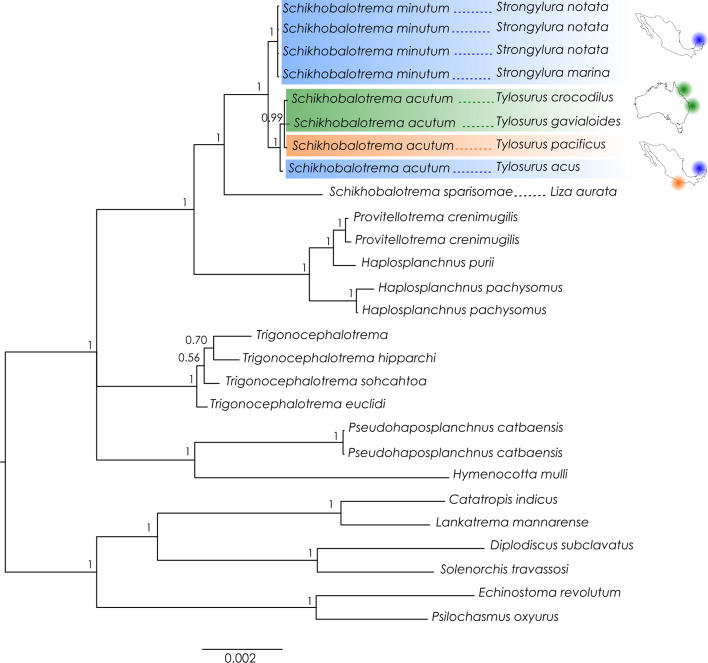
Relationships between members of the family Haplosplanchnidae inferred from BI analysis of the concatenated dataset (18S + 28S). *Schikhobalotrema* species along with host and sample site are also shown in the tree. Values at the nodes indicate posterior probabilities. GenBank accession numbers are shown in Table 1. Scale bar = number of substitutions per site.

Specimens from the intestines of needlefishes in 2 localities of the Pacific coast of Mexico and 2 localities off Yucatán in the GoM were initially morphologically identified as *Schikhobalotrema acutum*. Four specimens obtained from *Strongylura* spp., 1 specimen from *T. acus* and 4 specimens from *T. pacificus* were sequenced for all 3 molecular markers; the 28S alignment was 1094 bp, the 18S alignment was 1732 bp and the *cox*1 alignment was 475 bp. In the phylogenetic tree inferred with the concatenated dataset (18S + 28S), the family Haplosplanchnidae resolved as a monophyletic group, as did the genus *Schikhobalotrema* (Fig. 1); in both cases, relationships were strongly supported.

The type-species of the genus, *Sch. acutum*, collected from *T. acus* from the GoM and *T. pacificus* from off Chamela Bay and Barra de Coyuca, Acapulco on the Mexican Pacific coast formed a strongly supported clade with sequences of *Sch. huffmani* collected from *T. crocodilus* and *T. gavialoides* from Australia (Fig. 1). The genetic divergence values for the 2 ribosomal genes between *Sch. huffmani* from Australia and *Sch. acutum* from the EP and GoM were very low, just 1–5 base positions for 28S (0.1–0.3%) and 1–2 base positions for 18S (0.1%). The *cox*1 divergence among isolates of *Schikhobalotrema* occurring in species of *Tylosurus* across the same geographic range was 3.2–7.4% (15–32 base positions). Given the similarities in morphology, host and molecular data, we here consider *Sch. huffmani* a junior synonym of *Sch. acutum* (see Remarks section for *Sch. acutum*).

**Figure 2.**
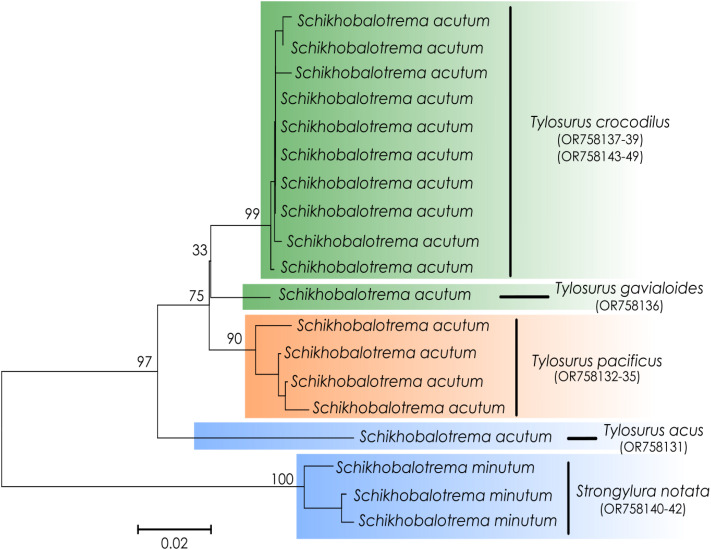
Phylogram of the NJ analysis of *cox*1 for species of *Schikhobalotrema*. Values at the nodes indicate posterior probabilities. GenBank accession numbers included after the species name. Scale bar = number of substitutions per site. Green colour refers to Australia; orange refers to the Pacific coast of Mexico; blue refers to GoM.

In both the 18S + 28S concatenated analysis and the *cox*1 analysis, *Sch. acutum* resolved as sister to a highly supported and reciprocally monophyletic clade comprising specimens of *Schikhobalotrema* collected from 2 species of *Strongylura* in La Carbonera coastal lagoon (Figs 1 and 2). This clade differed from *Sch. acutum* by 1–1.6% (8–13 base positions) in the 28S data, 0.1–0.2% (3–5 base positions) in the 18S data and 12.5–14.5% (56–65 base positions) in the *cox*1 data. Given the distinction in genetic data in sympatry (particularly the *cox*1 data), we consider this clade to represent a distinct species, which we describe as *Schikhobalotrema minutum* n. sp.

#### Family Haplosplanchnidae Poche, 1926 **Genus *Schikhobalotrema*** Skrjabin & Guschanskaja, 1955 ***Schikhobalotrema acutum*** (Linton, 1910) Skrjabin & Guschanskaja, 1955 (Figs 3B, C; 4B, C, D; 5B, C, D; 6B, C, D).

**Synonyms**: *Distomum* sp. of Linton (1907); *Deradena acuta* Linton, 1910; *Haplosplanchnus acutus* (Linton, 1910) Manter, 1937; *Schikhobalotrema huffmani* Huston, Cutmore & Cribb, 2017 (see Table 2).

**Figure 3.**
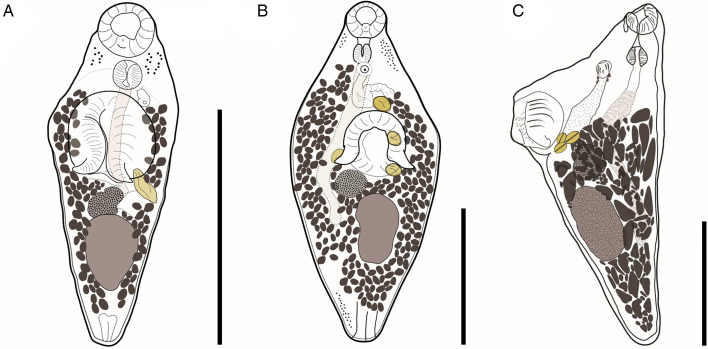
Line drawings of species of *Schikhobalotrema* from marine and estuarine fishes of Mexico: (A) *Schikhobalotrema minutum* n. sp. ex *Strongylura notata*, ventral view; (B) *Schikhobalotrema acutum* ex *Tylosurus acus*, ventral view and (C) *Sch. acutum* ex *Tylosurus pacificus*, lateral view. Scale bars = 500 *μ*m.

**Figure 4.**
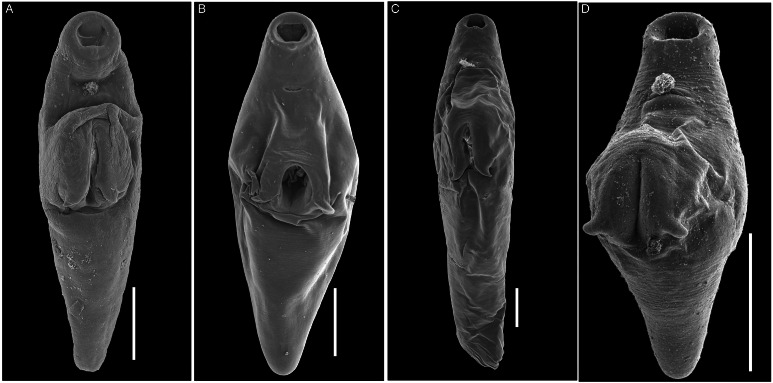
SEM photomicrographs of the entire body of 3 species of *Schikhobalotrema*: (A) *Sch. minutum* n. sp. ex *St. notata*, La Carbonera coastal lagoon, Yucatán, Mexico; (B) *Sch. acutum* ex *T. acus*, off Celestún, Yucatán, Mexico; (C) *Sch*. *acutum* ex *T*. *pacificus*, off Barra de Coyuca, Acapulco, Mexico and (D) *Sch. acutum* ex *Tylosurus crocodilus*, Lizard Island, Great Barrier Reef, Australia. Scale bars = 200 *μ*m.

**Figure 5.**
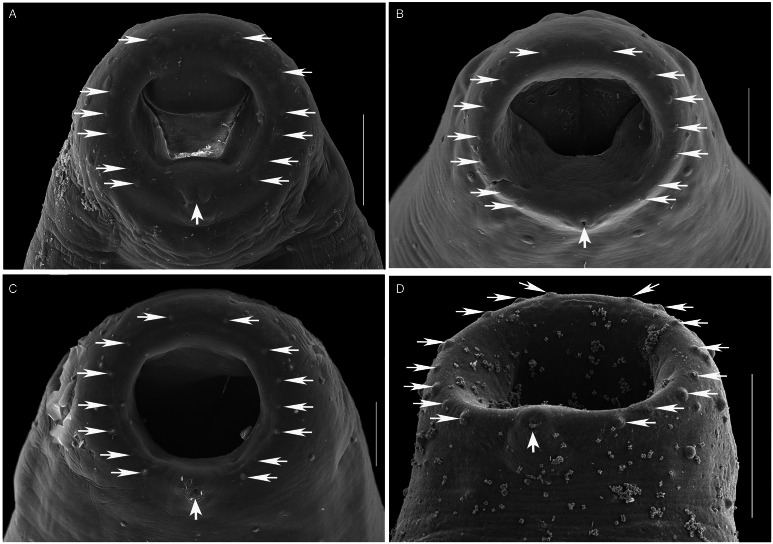
SEM photomicrographs of the oral sucker of 3 species of *Schikhobalotrema* showing the distribution of papillae: (A) *Sch. minutum* n. sp. ex *St. notata*, La Carbonera coastal lagoon, Yucatán, Mexico; (B) *Sch. acutum* ex *T. acus*, off Celestún, Yucatán, Mexico; (C) *Sch*. *acutum* ex *T. pacificus*, off Barra de Coyuca, Acapulco, Mexico and (D) *Sch. acutum* ex *T*. *crocodilus*, Lizard Island, Great Barrier Reef, Australia. Scale bars = 50 *μ*m.

**Figure 6.**
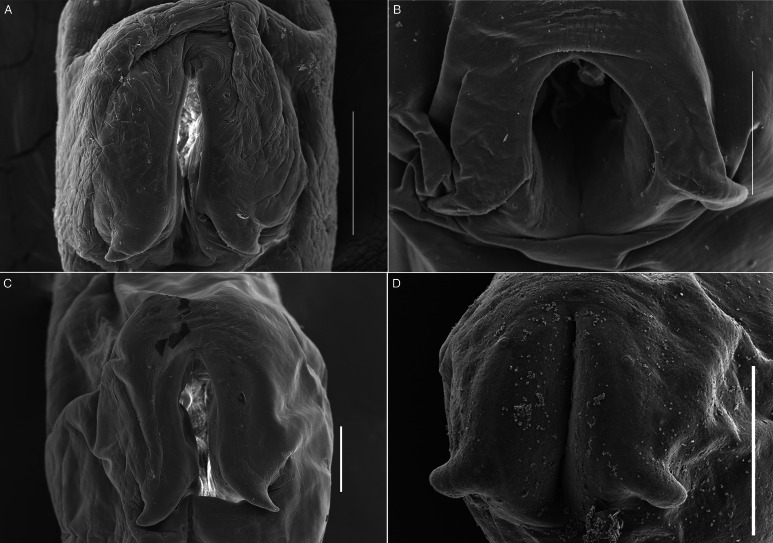
SEM photomicrographs of the ventral sucker of 3 species of *Schikhobalotrema* showing 2 lateral appendages on the posterior end: (A) *Sch. minutum* n. sp. ex *St. notata*, La Carbonera coastal lagoon, Yucatán, Mexico; (B) *Sch. acutum* ex *T. acus*, off Celestún, Yucatán, Mexico; (C) *Sch*. *acutum* ex *T. pacificus*, off Barra de Coyuca, Acapulco, Mexico and (D) *Sch. acutum* ex *T*. *crocodilus*, Lizard Island, Great Barrier Reef, Australia. Scale bars = 100 *μ*m.

**Table 2.** Measurements of some morphological traits of *Schikhobalotrema* species parasitizing belonids

	Present study (*n* = 19) *Sch. acutum* Chamela Jal.^a^ Lateral view	Present study (*n* = 4) *Sch. acutum* Acapulco, Gro. Lateral view	Huston *et al*. (2017) (*n* = 30) *Sch. acutum* Australia Lateral view	Caballero *et al*. (1953) (*n* = 2) *Sch. acutum* Panamá Lateral view	Manter (1938) (*n* = 4?) *Sch. acutum* Dry Tortugas, Florida Slightly lateral view	Present Study (*n* = 2) *Sch. acutum* Celestún, Yucatán Ventral view	Present Study (*n* = 6) *Schikhobalotrema minutum* n. sp. La Carbonera Lagoon, Yucatán Ventral view
BL	(647–1783) **1175**	(1068–1350) **1229**	(678–1023) **831**	1627–2058	1267–1485	(1203–1284) **1244**	(378–761) **545**
BW	(317–890) **534.3**	(518–714) **658.6**	(350–529) **424**	498–913	405–525	(574–596) **585**	(164–316) **223**
OSL	(78–192) **129.3**	(124–179) **149.7**	(90–126) **101**	106–114	150–172 (diameter)	(133–149) **141**	(76–134) **96**
OSW	(84–174) **115.6**	(136–195) **169.3**	(117–149) **132**	190–258		(160–166) **163**	(82–134) **108**
VSL	(152–332) **226.5**	(239–275) **262.5**	(205–274) **239**	531–614	262–337 (diameter)	(232–260) **246**	(131–207) **165**
VSW	(110–288) **173.5**	(205–266) **242.8**	(144–192) **166**	963–1119		(223–236) **229**	(94–197) **141**
PFL	(17–68) **44.5**	(35–82) **58.8**	(26–52) **40**	30–46	–	–	(23–70) **41**
PHL	(52–114) **73.4**	(104–135) **115.8**	(49–72) **61**	95–133	76–93	(74–89) **82**	(47–74) **63**
PHW	(48–109) **73.3**	(94–11) **108.9**	(69–95) **80**	122–152	70–87	(79–91) **85**	(48–66) **53**
OEL	(46–157) **95.8**	–	(52–87) **66**	95–133	Very short	–	(26–41) **35**
CL	(305–806) **461.6**	–	(256–387) **316**	–	–	(721–730) **710**	(250–290) **271**
TL	(167–437) **310.5**	(217–390) **320.9**	(167–288) **222**	432–448	–	(279–287) **282**	(80–185) **130**
TW	(109–289) **190.2**	(140–273) **221.2**	(118–184) **149**	266–365		(174–214) **194**	(60–140) **90**
OL	(69–157) **88**	–	(74–128) **100**	125–152	–	(100–114) **107**	(44–86) **70**
OW	(58–129) **84.5**		(68–101) **85**	122–247		(89–131) **110**	(33–84) **60**
EL	(42–87) **53.1**	(78–90) **82.8**	(61–85) **75**	87–45	73–85	(71–89) **77**	(61–90) **73**
EW	(21–46) **31**	(46–55) **48.9**	(42–58) **49**	57	51–56	(46–55) **49**	(38–57) **50**

B, Body; OS, oral sucker; VS, ventral sucker; PF, pre-pharynx; PH, pharynx; OE, oesophagus; C, caecum; T, testis; OV, ovary, E, egg.

Mean value in bold.

a Flattened specimens.

**Type-host**: *Strongylura marina* (Walbaum) (Beloniformes: Belonidae).

**Other hosts**: See Table 3.

**Table 3.** Reports of *Sch. acutum*

Host Order, Family Host species	Record reference	Detailed locality from reference	D	F	M
Atheriniformes: Atherinopsidae					
*Atherinella pachylepis* (Günther, 1864)	Sogandares-Bernal (1959)	Panama, Pacific coast, Panama City	No	No	No
Beloniformes: Belonidae					
*Platybelone argalus argalus* (Lesueur, 1821)	Nahhas and Cable (1964)	Curacao	No	No	No
*Strongylura incisa* (Valenciennes, 1846)	Machida and Kuramochi (2000)	Japan, Ryukyu Islands	Yes	No	No
*Strongylura marina* (Walbaum, 1792)	Kohn and Fernandes (1982)	Brazil, off Rio de Janeiro	No	**Yes**	No
*Strongylura marina* (Walbaum, 1792)	Nahhas and Short (1965)	Gulf of Mexico	No	No	No
*Strongylura marina* (Walbaum, 1792)	Linton (1910)	USA, Dry Tortugas, Florida	**Yes**	**Yes**	No
*Strongylura* sp.	Hanson (1950)	Bermuda	No	No	No
*Strongylura* sp.	Cable (1954)	Puerto Rico	**Yes**	**Yes**	No
*Strongylura* sp.	Siddiqi and Cable (1960)	Puerto Rico	No	**Yes**	No
*Strongylura timucu* (Walbaum, 1792)	Sosa-Medina *et al*. (2015)	Mexico, Atlantic coast, Yucatán, Celestún	No	No	No
*Strongylura timucu* (Walbaum, 1792)	Nahhas and Cable (1964)	Curacao	No	No	No
*Strongylura timucu* (Walbaum, 1792)	Overstreet (1969)	USA, Biscayne Bay, Florida	**Yes**	**Yes**	No
*Tylosurus acus* (Lacepède, 1803)	Rees (1970)	Bermuda	No	No	No
*Tylosurus acus* (Lacepède, 1803)	**Present study**	Mexico, Atlantic Coast, Yucatán, off Celestún	**Yes**	**Yes**	**Yes**
*Tylosurus choram* (Rüppell, 1837)	Zhukov (1977)	India, Madras region	**Yes**	**No**	**No**
*Tylosurus crocodilus* (Péron & Lesueur, 1821)	Madhavi (1979)	India, Bay of Bengal, Waltair coast	**Yes**	**Yes**	No
*Tylosurus crocodilus* (Péron & Lesueur, 1821)	Sogandares-Bernal (1959)	Bahamas, North Bimini	No	No	No
*Tylosurus crocodilus* (Péron & Lesueur, 1821)	Shen (1990)	China, Hainan Island	**Yes**	**Yes**	No
*Tylosurus crocodilus* (Péron & Lesueur, 1821)	Perez-Vigueras (1956)	Cuba	**Yes**	No	No
*Tylosurus crocodilus* (Péron & Lesueur, 1821)	Nahhas and Cable (1964)	Jamaica	No	No	No
*Tylosurus crocodilus* (Péron & Lesueur, 1821)	Machida and Kuramochi (2000)	Philippines, Mactan Island	**Yes**	No	No
*Tylosurus crocodilus* (Péron & Lesueur, 1821)	Manter (1937)	USA, Dry Tortugas, Florida	**Yes**	**Yes**	No
*Tylosurus crocodilus* (Péron & Lesueur, 1821)	Huston *et al*. (2017)	Australia, Queensland, GBR, Lizard Island	**Yes**	**Yes**	**Yes**
*Tylosurus crocodilus* (Péron & Lesueur, 1821)	Manter (1940)	Unclear: Galapagos Islands or Colombia	No	No	No
*Tylosurus crocodilus* (Péron & Lesueur, 1821)	Caballero *et al*. (1953)	Panama, Pacific coast, Fuerte Amador	**Yes**	**Yes**	No
*Tylosurus crocodilus* (Péron & Lesueur, 1821)	Manter (1940)	Colombia, Port Utria	No	No	No
*Tylosurus crocodilus* (Péron & Lesueur, 1821)	Manter (1947)	USA, Dry Tortugas, Florida	No	No	No
*Tylosurus gavialoides* (Castelnau, 1873)	Huston *et al*. (2017)	Australia, Queensland, Moreton Bay	**Yes**	**Yes**	**Yes**
*Tylosurus pacificus* (Steindachner, 1876)	**Present study**	Mexico, Pacific Coast, Jalisco, off Chamela Bay	**Yes**	**Yes**	**Yes**
*Tylosurus pacificus* (Steindachner, 1876)	**Present study**	Mexico, Pacific Coast, Guerrero, off Barra de Coyuca	**Yes**	**Yes**	**Yes**
Beloniformes: Hemiramphidae					
*Hemiramphus marginatus* (Forsskål, 1775)	Madhavi (1979)	India, Bay of Bengal, Waltair coast	**Yes**	**Yes**	No
*Hyporhamphus quoyi* (Valenciennes, 1847)	Machida and Kuramochi (2000)	Philippines, Palawan Island	**Yes**	No	No
*Hyporhamphus unifasciatus* (Ranzani, 1842)	Cable (1954)	Puerto Rico	**Yes**	**Yes**	No
*Hyporhamphus unifasciatus* (Ranzani, 1842)	Siddiqi and Cable (1960)	Puerto Rico	No	**Yes**	No
*Rhynchorhamphus georgii* (Valenciennes, 1847)	Madhavi (1979)	India, Bay of Bengal, Waltair coast	No	**Yes**	No
Centrarchiformes: Kyphosidae					
*Kyphosus elegans* (Peters, 1869)	Manter (1940)	Unclear: Galapagos Islands or Colombia	No	No	No
Eupercaria i.s.: Pomacentridae					
*Abudefduf saxatilis* (Linnaeus, 1758)	Sogandares-Bernal and Sogandares (1961)	Panama, Atlantic coast	No	**Yes**	No
Mugiliformes: Mugilidae					
*Mugil cephalus* Linnaeus, 1758	Fayek *et al*. (1990)	Mediterranean Sea, Port Said fish market	**Yes**	**Yes**	No
Ovalanteria i.s.: Labridae					
*Lachnolaimus maximus* (Walbaum, 1792)	Fischthal (1977)	Belize	No	No	No
Ovalanteria i.s.: Lutjanidae					
*Lutjanus analis* (Cuvier, 1828)	Fischthal and Nasir (1974)	Venezuela, Los Roques Island	No	No	No
Scombriformes: Trichiuridae					
*Trichiurus lepturus* Linnaeus, 1758	Fischthal and Nasir (1974)	Venezuela, Los Roques Island	No	No	No

D, description; F, figures; M, molecular data.

All host names updated to current interpretations. See Discussion for comments.

**Type-locality**: Dry Tortugas, Florida, Gulf of Mexico.

**Other localities**: See Table 3.

**New material examined**: 10 vouchers ex *T. pacificus* from off Chamela Bay, CNHE 12035; 6 vouchers ex *T. pacificus* from off Barra de Coyuca, Acapulco, CNHE 12036 and 1 voucher ex *T. acus* from off Celestún, Yucatán, CNHE 12034.

**Site in host**: Intestine.

**Prevalence of infection**: Three of 10 *T*. *pacificus*, Chamela Bay, Jalisco, Mexico; 4 of 6 *T*. *pacificus*, Barra de Coyuca, Acapulco, Guerrero, Mexico; 1 of 3 *T*. *acus*, Celestún, Yucatán, Mexico.

**Representative DNA sequences**: ex *T*. *pacificus*, 4 sequences from off Barra de Coyuca, Acapulco, Guerrero: 28S rDNA (OR753902–OR753904), 18S rDNA (OR753906) and *cox*1 (OR758132–OR758135); ex *T. acus* from off Celestún, Yucatán, 1 sequence: 28S rDNA (OR753897), 18S rDNA (OR753905) and *cox*1 (OR758131); ex *T. gavialoides*, 1 sequence from Moreton Bay: *cox*1 (OR758136); ex *T. crocodilus*, 10 sequences from off Lizard Island (OR758137–OR758139, OR758143–OR758149).

**Description**: Measurements are provided in Table 2. Our specimens were consistent with previous descriptions of this species by other authors (e.g. Linton, 1910; Manter, 1937, Caballero *et al*., 1953, Huston *et al*., 2017).

**Remarks**: We were unable to detect any qualitative morphological or morphometric differences between our specimens of this species from Mexico and those of *Sch*. *huffmani* from Australia. Additionally, both phylogenetic analyses resolve the Australian specimens of *Sch*. *huffmani* and specimens of *Sch*. *acutum* from the Pacific coast of Mexico as sister clades with high nodal support (Figs 1 and 2). The level of genetic difference between the Australian and Mexican specimens is relatively low; specimens from Australia differ from those from the Pacific coast of Mexico at just 13–21 base positions (3.1–4.6%) and from those from the GoM at 30–32 base positions (7.2–7.6%) in the *cox*1 dataset, which is the most variable marker analysed. Although Huston *et al*. (2017) noted morphological differences between their specimens and those of *Sch*. *acutum* from other studies, inclusion of the new specimens somewhat erodes these distinctions. Huston *et al*. (2017) noted that *Sch*. *huffmani* had a smaller body and thus smaller general features; however, some of the new material from Mexico studied here is much closer in size to those of *Sch*. *huffmani* than from other reports of *Sch*. *acutum*. Other morphological differences reported by Huston *et al*. (2017) between their specimens and *Sch*. *acutum* were based mainly on the description given by Manter (1937); most other descriptions of this species have largely lacked in finer detail. It seems likely that these differences would have been considered less significant if specimens from other previous studies were considered. Considering the evidence available (morphological, molecular and host specificity), we conclude that *Sch*. *huffmani* is best considered a synonym of *Sch*. *acutum*.

***Schikhobalotrema minutum*** n. sp. (Figs 3A, 4A, 5A, 6A).

**Type-host:** *Strongylura notata* (Beloniformes: Belonidae).

**Other hosts:** *Strongylura marina* (Beloniformes: Belonidae).

**Type-locality:** La Carbonera coastal lagoon (21°13′48.2″ N; 89°53′20.5″ W).

**Type-material:** Holotype (CNHE 12032) and 5 paratypes (CNHE 12033).

**Site in host:** Intestine.

**Prevalence of infection:** Three of 24 *St. notata* (12.5%); 1 of 2 *St. marina* (50%).

**Representative DNA sequences:** ex *St. notata* 3 sequences: 28S rDNA (OR753898–OR753899, OR753901), 18S rDNA (OR753907–OR753908, OR753910) and *cox*1 (OR758140–OR758142). ex *St. marina*, 1 replicate for each gene: 28S rDNA (OR753900) and 18S rDNA (OR753909).

**Etymology:** The specific epithet refers to the overall smaller body size of the new species compared with the congeneric species for which sequence data are available.

**ZooBank LSID:** urn:lsid:zoobank.org:pub:9982DE1C-90C5-45DF-B608-04AA79609C5F.

**Description** (Based on 6 dorso-ventrally mounted specimens, 5 ex *St. notata* and 1 ex *St. marina* from La Carbonera coastal lagoon and 1 specimen processed for SEM). Body small, tapering anteriorly and posteriorly, 378–761 × 164–316 (545 × 223) (Figs 3A and 4A). Tegument thin, lacking dorsal annulations. Eyespot pigment dispersed in forebody, around pharynx. Oral sucker round, 76–134 × 82–134 (96 × 108), bearing conspicuous frontal gland with a pore in ventral lip and 6 pairs of papillae arranged symmetrically (Fig. 5A). Ventral sucker large, larger than oral sucker, in midbody, with 2 conspicuous lateral appendages at posterior end, 131–207 × 94–197 (165 × 141) not considering lobe extensions; aperture a longitudinal slit (Figs 3A, 4A and 6A). Ventral sucker/oral sucker length ratio 1.3–2.2 (1.7):1; ventral sucker/oral sucker width ratio 1.1–1.5 (1.3):1. Forebody 115–240 (175), occupying 30–33 (32%) of body length. Prepharynx distinct, 23–70 (41). Pharynx ovoid, 47–74 × 48–66 (63 × 53). Oesophagus inconspicuous, 26–41 (35) long. Caecum extending dorsally to level with anterior margin of ovary.

Testis singular, in mid-posterior third of body, transversally elongate, 80–185 × 60–140 (130 × 90), occupying 24–25 (25%) of body length. Seminal vesicle short, tubular, forming a globular prostatic bulb before opening at genital atrium. Genital pore ventral, lateral to pharynx. Ovary irregularly shaped, slightly overlapping anterior portion of testis, 44–86 × 33–84 (70 × 60). Laurer's canal and Mehlis gland not observed. Seminal receptacle globular, lateral to ovary. Vitellarium follicular, in 2 lateral fields extending from anterior margin of ventral sucker to mid-region between posterior margin of testis and posterior extremity; fields apparently not confluent. Uterus passes ventral sucker dorsally. Eggs large, 61–90 × 38–57 (73 × 50). Excretory vesicle I-shaped. Excretory pore terminal.

**Remarks**: In the possession of a ventral sucker with 1 pair of lateral appendages on the posterior margin and a longitudinal aperture, the new species is similar to 3 species of *Schikhobalotrema*: *Sch. acutum*, *Schikhobalotrema ablennis* (Abdul-Salam and Khalil, 1987) Madhavi, 2005 and *Schikhobalotrema adacutum* (Manter, 1937) Skrjabin and Guschanskaja, 1955. The new species differs from *Sch. ablennis* in having a more anterior testis and a shorter forebody. Further, *Sch. ablennis* was reported from a different genus of belonid *Ablennes* Jordan & Fordice (see Abdul-Salam and Khalil, 1987), whereas the new species was found in *Strongylura* spp. The new species differs from *Sch. adacutum* in having a more conspicuous lateral appendage of the ventral sucker, and even though both are found in the GoM, *Sch. adacutum* is known from species in the families Atherinidae, Hemiramphidae, Labridae, Scaridae and Pomacentridae, and not members of the Belonidae.

Morphologically the new species most closely resembles *Sch. acutum* which is also reported from belonids. These 2 species share the presence of a conspicuous frontal gland in the ventral lip of the oral sucker but are distinct in that there are 6 pairs of papillae symmetrically arranged around the oral sucker in the new species, *vs* 7–8 pairs for *Sch. acutum* (Fig. 5). The new species further differs from *Sch. acutum* by the possession of a caecum that extends to level with the ovary, *vs* to level with the middle portion of the testis in *Sch. acutum* (Fig. 3). Further, the new species differs in the overall body size (it is distinctly smaller), and thus possesses distinctly smaller oral and ventral suckers, pharynx and genitalia (Table 2, Fig. 3). Finally, the new species is clearly genetically distinct from *Sch. acutum* for all 3 gene regions analysed in this study.

## Discussion

### Trematode identification over geographical range

Recently there have been numerous studies using *cox*1 data to explore the delimitation of marine fish trematodes over wide geographic ranges in the IWP. Huston *et al*. (2021) tested species boundaries within the gorgocephalid genus *Gorgocephalus* Manter, 1966 in the IWP, demonstrating that 3 of the 4 *Gorgocephalus* species studied had convincingly wide distributions in the region, with *Gorgocephalus yaaji* distributed across the IWP extremes (South Africa to French Polynesia). Cutmore *et al*. (2021) reported that some species of blood flukes (Aporocotylidae) are widely distributed in the IWP, with *cox*1 data indicating that *Ankistromeces olsoni* Nolan & Cribb, 2006 is found from Australia to Japan and *Phthinomita sasali* Nolan & Cribb, 2006 is found from Ningaloo Reef in the Indian Ocean to Palau in the Pacific Ocean. Cutmore and Cribb (2022) demonstrated that the blood fluke *Elaphrobates chaetodontis* (Yamaguti, 1970) Yong, Cribb & Cutmore, 2021 is similarly widespread, from *cox*1 data from Australia, Japan and French Polynesia forming a well-supported and geographically structured clade. Wee *et al*. (2022) demonstrated that *Helicometroides longicollis* Yamaguti, 1934 (Monorchiidae) is distributed between Japan and Australia, and Cribb *et al*. (2022) showed that *Bivesicula claviformis* Yamaguti, 1934 is found, at least, in Japan and of both the Indian and Pacific coasts of Australia. It must be noted, however, that for each of these widespread species, all these above studies demonstrated that at least some of their congeners were highly restricted with equally convincing data.

Most notably, Bray *et al*. (2022) studied species of the lepocreadiid genus *Preptetos* Pritchard, 1960 infecting acanthurid fishes from a range of sites in the IWP. In this study, the authors proposed a set of objective criteria for the recognition of trematode species, with as a first step the reciprocal monophyly using the most discriminating available molecular marker, and at least one of the following criteria: consistent morphological differences relative to other species, or consistent differences in host distribution with respect to close relatives. In our study, we followed the paradigm proposed by Bray *et al*. (2022) analysing the morphological and molecular variation, host association (host-specificity), the historical biogeography of the host group, the habitat of the host and the geographical distribution of both associates to determine whether the distinct genetic lineages correspond to separate species of *Schikhobalotrema*.

The new species, *Sch. minutum* n. sp. was resolved as a reciprocally monophyletic group in both the 18S + 28S rDNA and *cox*1 mtDNA phylogenetic analyses (Figs 1 and 2). The new species differed from *Sch. acutum* at 56–65 base positions (12.5–14.5%) in the *cox*1 dataset, a difference consistent with the recognition of closely related but distinct species for a range of trematode families in the IWP (e.g. Cutmore *et al*., 2021, 2023; Huston *et al*., 2021; Bray *et al*., 2023; Magro *et al*., 2023). Further, for *Sch. minutum* n. sp. we observed some morphological characters to differentiate the new species, mostly based on body size and the extension of the caecum in the body. Relative to the paradigm proposed by Bray *et al*. (2022), these traits support the recognition of a new species.

Interpretation of the new specimens relating to *Sch. acutum* relative to the description of *Sch. huffmani* is more complex, with reports from across the Pacific and Atlantic oceans. *Schikhobalotrema acutum* was originally described from the GoM, but it has since been reported from the Caribbean Sea, Brazil, Colombia, the Galapagos Islands, Panama, India, Japan and the Philippines (Manter, 1940; Caballero *et al*., 1953; Siddiqi and Cable, 1960; Madhavi, 1979; Machida and Kuramochi, 2000; Kohn *et al*., 2007). Although molecular data are not available for samples from most of the host records, we now have *cox*1 data for samples from 4 marine realms, the tropical Atlantic, tropical EP, the central Indo-Pacific and temperate Australia (see Spalding *et al*., 2007). These data demonstrate that populations of *Sch. acutum* across a wide geographic range have only small *cox*1 divergence values; new samples from the GoM and the Pacific coast of Mexico differ at just 30–34 base positions (7.2–7.9%), and those from the Pacific coast of Mexico and Australia differ at just 13–21 base positions (3.1–4.6%). These values, and the lack of divergence for both ribosomal genes, support the interpretation of a single species across these regions. These divergence values generally agree with those obtained for other marine fish trematode genera. McNamara *et al*. (2014) tested the identity of 16 morphospecies of *Hurleytrematoides* Yamaguti, 1954 parasitizing a wide range of Chaetodontidae species in the IWP. They recognized species boundaries at a minimum of 55 base positions (9.1%) in *cox*1. Confidence in these interpretations was supported by the fact that the morphospecies of *Hurleytrematoides* show clear distinctions in their complex terminal genitalia, contrasting with the general morphological similarity of many combinations of haplosplanchnid species. Increasingly, however, studies are demonstrating the propensity for cryptic speciation in the Trematoda which seems to be a pattern as suggested by Pérez-Ponce de León and Poulin (2018), and morphological differences supporting genotypic distinctions are not always evident. As for the studies by Huston *et al*. (2021), Bray *et al*. (2022), Cribb *et al*. (2022) and Cutmore *et al*. (2021), the hypothesis we present herein for *Schikhobalotrema* is based heavily on the interpretation of the *cox*1 data; although partially supportive of the *cox*1 relationships, ribosomal data are proving to be less informative to delineate species in trematodes that have conserved morphology, but still a proper marker to separate species (see Pérez-Ponce de León and Hernández-Mena, 2019). Genetic data are not available for most of the species in the genus *Schikhobalotrema*; sequence data have been generated for just 4 of the 27 valid species. Clearly, genetic information for more congeneric species is needed to achieve robust conclusions regarding patterns of geographical distribution and host-specificity in this trematode genus. DNA information, particularly that from the most variable marker, the mitochondrial *cox*1, proved critical for drawing conclusions in the current study.

### Biogeography

In this study, we provide molecular and morphological data in support of the hypothesis that *Sch. acutum* is distributed across a wide geographic range incorporating the GoM, the western Pacific and the EP. Some marine organisms, particularly certain fishes, can readily disperse over great distances. For instance, it has been shown that some species may pass what Lessios and Robertson (2006) considered the ‘impassable’ eastern Pacific barrier, ca. 5000 km of deep water that separates the eastern from the central Pacific which is the widest marine biogeographic barrier in the world (Lessios and Robertson, 2006). While there have been no molecular studies published regarding broad distributions of the belonid genus *Tylosurus*, some species are known to have wide ranges; according to FishBase the hound needlefish, *T. crocodilus*, is distributed in the IWP, the tropical Atlantic, and off the coasts of Africa and the Americas. Wide host ranges of fishes are, however, not necessarily mirrored by their trematode parasites; due to the complex life cycles of trematodes and the absence of long-lived dispersive larval stages, they may have far more restricted ranges. There are, however, some notable exceptions for those species infecting highly vagile marine hosts, such as the blood flukes infecting bluefin tuna (Aiken *et al*., 2007) and spirorchiid blood flukes of marine turtles (Corner *et al*., 2022, 2023). Our current interpretation of the available sequence data for specimens morphologically consistent with *Sch. acutum* indicates that it has an exceptional distribution, occurring from Australia to the Mexican Pacific coast and the GoM. Molecular characterization of samples from other reported localities and other reported belonid hosts (*Tylosurus fodiator*, *Strongylura incisa*, *St. marina* and *Strongylura timucu*) will doubtless further improve understanding of this system.

While it appears that a close phylogenetic relationship between hosts and parasites, and some life-history traits of the host, seem to explain the large geographic range exhibited by *Sch. acutum*, more sequence data and the analysis of other trematode species and even other parasite taxa such as monogeneans and copepods (which also seem to exhibit a strong host-specificity towards belonids) will prove useful for understanding the historical biogeography of belonids and their parasitic fauna.

### Host-specificity

Table 3 summarizes host reports of *Sch. acutum*, including the 3 new records made here and interpreting the original reports of *Sch. huffmani* as *Sch. acutum*. Multiple higher taxa of fishes are involved, but the Beloniformes account for well over half the reports. In our view, none of the reports from other orders of fishes is strongly credible given the general lack of evidence provided and the rarity of the combinations: the single record from an atherinopsid by Sogandares-Bernal (1959) was considered ‘accidental’ in the original report and lacked evidence; the report from a kyphosid was of a single specimen (Manter, 1940) and lacked evidence; the report from a labrid was of 11 individual worms (Fischthal, 1977) but lacked evidence; the report from a lutjanid was of 3 specimens (Fischthal and Nasir, 1974) but lacked evidence; the report from a mugilid by Fayek *et al*. (1990) can be unambiguously discounted on the basis that the ventral sucker is shown as lacking processes and the eggs are embryonated with miracidia; the report from pomacentrid has an image consistent with *Sch. acutum* but was of a non-gravid specimen (Sogandares-Bernal and Sogandares, 1961) and the report from trichiurid was of 2 worms (Fischthal and Nasir, 1974) but lacked evidence. It seems unlikely that any of these fishes represent regular hosts for *Sch. acutum* and certainly all need further verification.

Infections of *Sch. acutum* are clearly concentrated in beloniforms and, among them, in belonids (although many of these reports also lack evidence). However, 4 studies have reported infections from hemiramphids. Cable (1954), in work directed at the first elucidation of a haplosplanchnid life cycle, reported that *Sch. acutum* was ‘common in needle-fish and half-beaks’ in his study area. Siddiqi and Cable (1960) reported *Sch. acutum* from both a belonid and a hemiramphid but without any prevalence details. Madhavi (1979) reported *Sch. acutum* from both a belonid and 2 hemiramphids but did not report prevalence data and her figure did not indicate the host of the sample. Machida and Kuramochi (2000) reported a single specimen of *Sch. acutum* from a hemiramphid together with 4 from belonids; their descriptions did not distinguish between specimens from the 2 families. Given the expertise of the workers involved the reports seem broadly credible, but we consider the issue of whether *Sch. acutum* is genuinely shared by both belonids and hemiramphids to be unresolved. In favour of the sharing are the repeated reports by multiple experts together with the fact that the 2 fish families belong to the same order of fishes and occupy similar habitats. Against the sharing is the lack of positive evidence (descriptions, figures, molecular data) of the infections in hemiramphids and the fact that, although related, belonids and hemiramphids have dramatically differing diets. Belonids are overwhelmingly piscivores whereas hemiramphids are omnivorous, eating mainly algae and invertebrates. In this context we note that we have examined 237 hemiramphid individuals from Australian localities where infection in belonids were detected but have found no infections of *Sch. acutum*; a similar situation holds true for Mexican localities of both the Pacific and the GoM coasts where we have examined around 130 hemiramphids, in which *Sch. acutum* has not been found.

The presence of haplosplanchnids in belonids is essentially unexplained. What is known of haplosplanchnid life cycles suggests that typically their cercariae encyst in the open (Cable, 1954; Fares and Maillard, 1975) probably typically in association with algae. This form of transmission is consistent with their concentration in herbivorous fishes. According to our records, just 4 fish families (Acanthuridae, Labridae [overwhelmingly the subfamily Scarinae], Mugilidae and Pomacentridae), which all incorporate significant grazing of algae, account for over 80% of the host records for haplosplanchnids. No family of piscivores other than the Belonidae is significant as hosts. Given the presence of species of *Schikhobalotrema* with paired appendages on the ventral sucker in both belonids and hemiramphids, we predict that infections in belonids arose as a host switch into the latter family. However, the mode of transmission remains unknown.

## Data Availability

Sequence data are available in the NCBI GenBank database.
